# Cannabinoid receptor CB1 mediates baseline and activity-induced survival of new neurons in adult hippocampal neurogenesis

**DOI:** 10.1186/1478-811X-8-12

**Published:** 2010-06-17

**Authors:** Susanne A Wolf, Anika Bick-Sander, Klaus Fabel, Perla Leal-Galicia, Svantje Tauber, Gerardo Ramirez-Rodriguez, Anke Müller, Andre Melnik, Tim P Waltinger, Oliver Ullrich, Gerd Kempermann

**Affiliations:** 1 Max Delbrück Center for Molecular Medicine (MDC) Berlin-Buch, and Volkswagenstiftung Research Group, Department of Experimental Neurology, Charité University Medicine, Berlin, Germany; 2 Institute of Anatomy, University of Zurich, Zurich, Switzerland; 3 National Institute of Psychiatry "Ramón de la Fuente Muñiz", Neuropharmacology Department, Calz. México-Xochimilco 101, 14370 México, D.F. México; 4 CRTD - Center for Regenerative Therapies Dresden, Dresden, Germany

## Abstract

**Background:**

Adult neurogenesis is a particular example of brain plasticity that is partially modulated by the endocannabinoid system. Whereas the impact of synthetic cannabinoids on the neuronal progenitor cells has been described, there has been lack of information about the action of plant-derived extracts on neurogenesis. Therefore we here focused on the effects of Δ9-tetrahydrocannabinol (THC) and Cannabidiol (CBD) fed to female C57Bl/6 and Nestin-GFP-reporter mice on proliferation and maturation of neuronal progenitor cells and spatial learning performance. In addition we used cannabinoid receptor 1 (CB1) deficient mice and treatment with CB1 antagonist AM251 in Nestin-GFP-reporter mice to investigate the role of the CB1 receptor in adult neurogenesis in detail.

**Results:**

THC and CBD differed in their effects on spatial learning and adult neurogenesis. CBD did not impair learning but increased adult neurogenesis, whereas THC reduced learning without affecting adult neurogenesis. We found the neurogenic effect of CBD to be dependent on the CB1 receptor, which is expressed over the whole dentate gyrus. Similarly, the neurogenic effect of environmental enrichment and voluntary wheel running depends on the presence of the CB1 receptor. We found that in the absence of CB1 receptors, cell proliferation was increased and neuronal differentiation reduced, which could be related to CB1 receptor mediated signaling in Doublecortin (DCX)-expressing intermediate progenitor cells.

**Conclusion:**

CB1 affected the stages of adult neurogenesis that involve intermediate highly proliferative progenitor cells and the survival and maturation of new neurons. The pro-neurogenic effects of CBD might explain some of the positive therapeutic features of CBD-based compounds.

## Background

The recreational use of cannabis is often justified by extrapolation from the unquestionable physiological role of endocannabinoids in brain function [1], and the successful and beneficial manipulation of the endocannabinoid system for medical purposes [2,3] by plant extracts from *cannabis sativa *or synthetic agonist and antagonists specific for cannabinoid receptor1 or 2 (CB1, CB2) [4,5]. The abuse of cannabis can be associated with detrimental long-term consequences, for example an increased risk of developing memory impairments [6,7].

The process of generating new neurons throughout life in the hippocampus probably plays a role in learning and memory processes [8], and impairment of adult hippocampal neurogenesis is thought to be part of the pathogenesis of neurodegenerative disorders like dementia, epilepsy and schizophrenia [9,10]. Adult neurogenesis is a particular example of brain plasticity as it involves the integration of entire cells [11,12]. Due to its physiological role in brain plasticity the endocannabinoid system might contribute to the control of adult hippocampal neurogenesis in health and disease. A number of arguments point into the direction that cannabinoids might exert some of their actions via their effects on adult neurogenesis (reviewed in [13]).

The therapeutic activities of cannabinoids include analgesia, immuno-suppression, mood stabilization, anti-emesis, bronchodilatation and neuroprotection [14]. Because of the psychotropic effects of some cannabinoids, their clinical use is limited. Cannabidiol (CBD) is the main non-psychotropic compound of the plant *cannabis sativa *and belongs to the group of exogenous cannabinoids [15]. Due to its lack of psychoactive actions, CBD represents one of the most promising candidates for clinical application [14]. CBD was shown to act anti-psychotic in Parkinson's disease and as a monotherapy in treatment-resistant schizophrenia [16,17]. The neuroprotective effects of CBD have been linked with its antioxidant activity [18]. Evidence emerges that CBD realizes some of its effect via the classical CB receptors [19].

Many constituents of the endogenous cannabinoid system like the CB1 and CB2 receptors and their endogenous ligands Anandamide (AEA) and 2-arachidonylglycerol (2-AG) as well as the AEA-degrading enzyme fatty acid amide hydrolase (FAAH) and the 2-AG synthesizing enzyme diacylglycerol lipases are found in neuronal developmental and adult neurogenesis [20-22].

Several studies investigating the role of the cannabinoid system in adult neurogenesis found that stimulation of CB1 seemed to either increase or decrease adult neurogenesis [21,23]. For example, the synthetic agonist HU210 decreased the number of intermediate progenitor cells in one study [24], but promoted neuronal differentiation in another [25]. In other studies, CB1 receptor activation promoted precursor cell proliferation and the generation of neurospheres ex vivo, which was abrogated in CB1-deficient precursor cells, and proliferation of hippocampal precursor cells was increased in FAAH deficient mice [21,23,26]. Likewise, in adult CB1-deficient mice, neural progenitor proliferation is decreased. In addition, endocannabinoid signaling controls neural progenitor differentiation in the adult brain by promoting astroglial differentiation of newly born cells [23]. Along the same line, Rueda et al. have shown that the endocannabinoid AEA inhibited neuronal progenitor cell differentiation through attenuation of the extracellular signal regulated kinase pathway in vitro, and that adult neurogenesis in the dentate gyrus was significantly decreased by the AEA analogue methanandamide and increased by the CB1 antagonist SR141716 [27].

Precursor cell proliferation is a relatively non-specific measure of neurogenesis and not identical to the net production of new neurons. Progenitor cell proliferation is, for example, increased after epileptic seizures without necessarily leading to functional neurogenesis [28]. The incorporation of the progenitor cell into the neuronal network is impaired after seizures despite a high proliferation rate [29]. In the study by Jin et al. only BrdU incorporation was measured without further phenotyping the labeled cells and only cell proliferation was directly addressed [30]. However, they reported increased cell proliferation after treatment with CB1 antagonists SR141716 and AM251, which is in line with the findings by Rueda at al. [27]. In addition, they showed that SR141716 enhances cell proliferation via the vanillin receptor 1 [30]. The absence of the CB1 receptor resulted again in decreased proliferation.

The confusion that emerges when comparing the studies could be explained by differences in the study design, compounds used, sex of the animals, duration of application, and the readout parameters for "adult neurogenesis". Moreover, in the context of adult neurogenesis, only synthetic compounds interacting with the endogenous cannabinoid system have been investigated so far. It has been speculated that also plant-derived cannabinoids might have an impact on neurogenesis, but no data exist to date. The sole exception is a brief study reporting no effects on cell proliferation in general [31].

In our study we therefore first examined the effects on adult neurogenesis in female C57Bl/6 mice by pharmaceutical extracts enriched with Δ9-tetrahydrocannabinol (THC) or Cannabidiol (CBD) directly derived from the plant *cannabis sativa*. Since THC's and CBD's mode of actions are partly CB1-dependent [19,32], we then looked at the time course (including proliferation and net-neurogenesis) of the maturation process of neuronal precursor cells in CB1-/- C57Bl/6 female mice and the impact of the CB1 antagonist AM251.

## Results

### Chronic THC treatment impairs spatial learning

**Figure 1:**
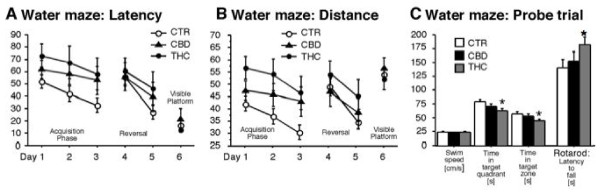
**Spatial learning was impaired after THC treatment**. C57Bl/6 female mice were either fed with food supplemented with THC-rich or CBD-rich plant extracts or a control diet. Spatial memory was tested after 6 weeks of treatment. THC mice were slower in finding the hidden platform over the whole acquisition period and during the reversal learning, with regard to both latency (A) and distance (B) to platform. All three groups performed at the same level when the platform was made visible on day 6 to test for possible visual impairments and the general ability to perform the task. A Rotarod test to measure general locomotor function and fitness was performed on day 7. The THC group performed better than CTR (C); * p ≤ 0.05.

Either THC- or CBD-enriched or control (CTR) diet was fed to female C57Bl/6 or Nestin-GFP-reporter mice. The food intake and the weight gain over the period of 6 weeks were similar in all the treatment groups (see additional file 1, Additional file 2). To examine the impact of chronic THC vs. CBD treatment on spatial memory, we tested the three experimental groups (THC, CBD, CTR) in the Morris water maze (MWM). The task in the MWM is to navigate to a hidden platform using spatial cues in the room. As shown in figure 1A, THC mice were slower in finding the hidden platform over the whole acquisition period (repeated measures ANOVA, F_2,20 _= 3.49; p = 0.0014). In addition, THC mice showed a significantly impaired performance during the reversal learning (with the hidden platform at a new position) with regard to both latency (THC: 36.13 +/- 10.94, CTR: 16.88 +/- 4.21, p = 0.002, ANOVA, F_2,20 _= 3.49, Fig. 1A) and distance to platform (THC: 49.4 ± 6.76, CTR: 31.93 ± 2.94, p = 0.002, ANOVA, F_2,20 _= 3.49; Fig. 1B). All three groups performed at the same level when the platform was made visible on day 6 to test for possible visual impairments and the general ability to perform the task (Fig. 1A, B). The impaired learning performance of the THC-treated mice was also reflected by the shorter time the animals spent in the old target quadrant and target zone during the probe trial at day 4 (Fig. 1C). A rotarod test to assess general locomotor functions and fitness was performed on day 7. The THC group performed better than CTR (CTR 138.47 ± 25.844 s, THC group 180.82 ± 26.17 s; p = 0.0046, ANOVA, F_2,16 _= 3.634; Fig. 1C), whereas CBD performed at control level. Therefore, the decreased performance of THC mice in the water maze could not be attributed to a reduced general fitness.

CBD mice showed some (statistically not significant, p = 0.124; F_2,20 _= 3.49; Fig. 1A, B) impairment during acquisition. During the probe trial (Fig. 1C) and the reversal they performed very similar to CTR.

### Chronic THC treatment decreases adult neurogenesis

We have previously reported that adult hippocampal neurogenesis can be linked to aspects of the acquisition phase during water maze learning [33] with a particular contribution to reversal performance [34,35]. We did not find a specific reversal phenotype in the present study but nevertheless asked whether a decrease in adult neurogenesis, possibly matching the observed alterations in water maze performance, might be found after THC or CBD treatment.

**Figure 2:**
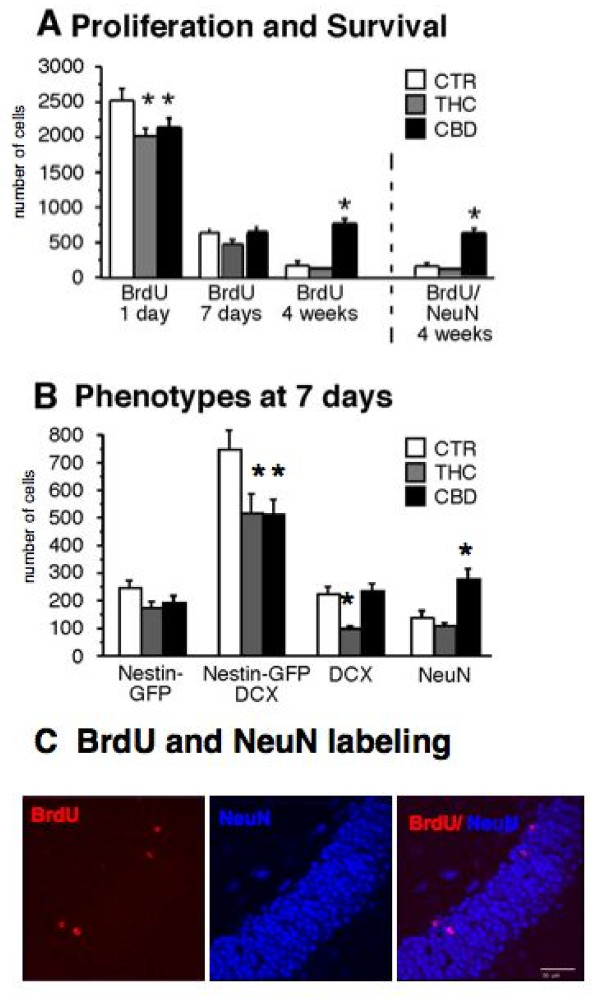
**CBD treatment enhanced adult neurogenesis**. BrdU cells reflect the population of proliferating cells at a given period of time. Proliferation was measured 24 h after BrdU injection, while survival was measured 4 weeks after BrdU injection. NeuN/BrdU double positive cells at 4 weeks after BrdU injection are the neurons that were generated and survived during the period of 4 weeks. THC treatment for 6 weeks reduced cell proliferation without affecting neuronal survival. In contrast, CBD treatment decreased proliferation as well, but increased neuronal cell survival seen at 4 weeks after BrdU injection. (A). Animals expressing Nestin, an early marker of neuronal maturation, under a GFP promotor were also fed with THC-rich, CBD-rich, or control (CTR) diet for 6 weeks. The animals were assessed at 7 days after BrdU injection to evaluate the early stages of neurogenesis. In both THC and CBD groups we found a minimal reduction in the number of BrdU-labeled type-1/2a cells (Nestin-GFP-positive, DCX-negative) but a significant reduction on the level of the type-2b (Nestin-GFP-positive, DCX-positive). In THC the number of DCX-positive/Nestin-GFP-negative cells was also reduced. There was a significant increase in the production of BrdU/NeuN-positive cells in the CBD group (B); * p ≤ 0.05. In (C) we show a representative micrograph of BrdU labeled cells (red) within the granule cell layer labeled with NeuN (blue) of the dentate gyrus. The section is one out of nine throughout one hippocampus of a female C57Bl/6 animal that received 3 BrdU injections 4 weeks prior to analysis. The section is 40 μm thick and the scale bar is 50 μm.

We found that chronic THC application reduced precursor cell proliferation in the DG (THC vs. CTR: 2018 ± 96; 2515 ± 180; n = 5; p = 0.037, Fig. 2A) without affecting cell survival or net neurogenesis. In contrast, however, despite decreasing proliferation, CBD increased cell survival (proliferation: CBD vs. CTR: 2083 ± 102 vs. 2515 ± 180; n = 5; p = 0.0358; survival: CBD vs. CTR: 756 ± 28 vs.180 ± 21; n = 5; p = 0.0012; Fig. 2A).

In both, THC and CBD groups we found a minimal reduction in the number of BrdU-labeled type-1/2a cells, i.e. Nestin-GFP-positive, Doublecortin (DCX)-negative cells, but a significant reduction at the level of the type-2b cells (Nestin-GFP-positive, DCX-positive; THC vs. CTR: 502 ± 83 vs. 748 ± 78; n = 5; p = 0.028; CBD vs. CTR: 507 ± 62 vs. 748 ± 78; n = 5; p = 0.032; Fig. 2B). In THC the number of DCX-positive/Nestin-GFP-negative cells was also reduced (THC vs. CTR: 113 ± 19 vs. 249 ± 26; n = 5; p = 0.004; Fig. 2B), possibly indicating that THC might not increase net neurogenesis but nevertheless accelerate the transition through the DCX-positive stage. For an overview of the maturation stages see below. A representative BrdU/NeuN staining is shown in figure 2C.

There was, however, a significant increase in the production of BrdU/NeuN-positive cells in the CBD group (CBD vs. CTR: 297 ± 32 vs.146 ± 23; n = 5; p = 0.001; Fig. 2A, B), suggesting that in this case an accelerated transition through the DCX stage might result in more neurons, an effect absent in the case of THC.

Taken together THC treated mice showed reduced water maze performance, albeit not the presumably neurogenesis-related reversal impairment, and reduced adult neurogenesis, whereas CBD mice did not. On the other hand our results pointed to a positive effect of CBD on adult neurogenesis that we now intended to explore further.

### CBD effects on adult neurogenesis are absent in CB1 -/- mice

**Figure 3:**
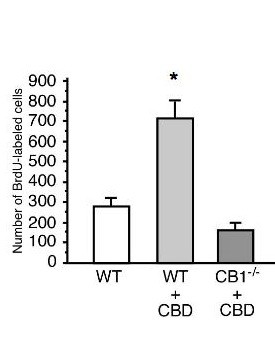
**CBD effect was absent in CB1-/- mice**. We fed additional wild type and CB1^-/- ^mice with CBD for 6 weeks. We found that the increase in cell survival induced by CBD was abolished in CB1^-/- ^mice; * p ≤ 0.05.

We next asked whether these CBD-mediated effects on BrdU incorporation might be mediated by the CB1 receptor, which is highly expressed in the dentate gyrus and fed CBD to CB1-/- mice and their wild type litter mates in parallel for 6 weeks. We found that the increase in BrdU cell survival induced by CBD was abolished in CB1-/- mice (Fig. 3; WT/CBD vs. CB1-/-/CBD: 716 ± 83 vs.152 ± 28; n = 5; p = 0.001). CB1-/- mice showed a decrease in the number of BrdU cells. This decrease in CB1-/- has already been described in the literature. But we could here show a similar effect of CB1-/- at the survival time point at 4 weeks after BrdU application (CB1-/- vs. WT: 180 ± 15 vs.368 ± 21; n = 5; p = 0.002).

### CB1 is expressed during the DCX-stage of adult hippocampal neurogenesis

Given this relevance of the CB1 receptor for the observed adult neurogenesis phenotype we next asked, which cells would express the CB1 receptor in the course of adult neurogenesis. We used hippocampal sections from untreated female Nestin-GFP-reporter mice. Based on these mice we have previously proposed a model of neuronal development in the adult hippocampus [36-38]. From a Nestin-GFP-positive radial glia-like putative stem cell (type-1) development proceeds over a population of highly proliferative Nestin-GFP-positive intermediate progenitor cells with glial properties, but lacking the radial morphology (type-2a) and a similarly proliferative progenitor cell which is still Nestin-GFP-positive but also expresses DCX (as well as, for example, Prox1 and NeuroD1) and thus shows signs of neuronal determination (type-2b). Migratory, neuroblast-like type-3 cells are DCX-positive but do not express Nestin-GFP anymore. They show limited proliferation. After this stage, cells go through a postmitotic maturation stage, during which the new neurons extend their neurites and which is associated with the transient expression of Calretinin [37,39] and of the lasting postmitotic neuronal marker NeuN.

**Figure 4:**
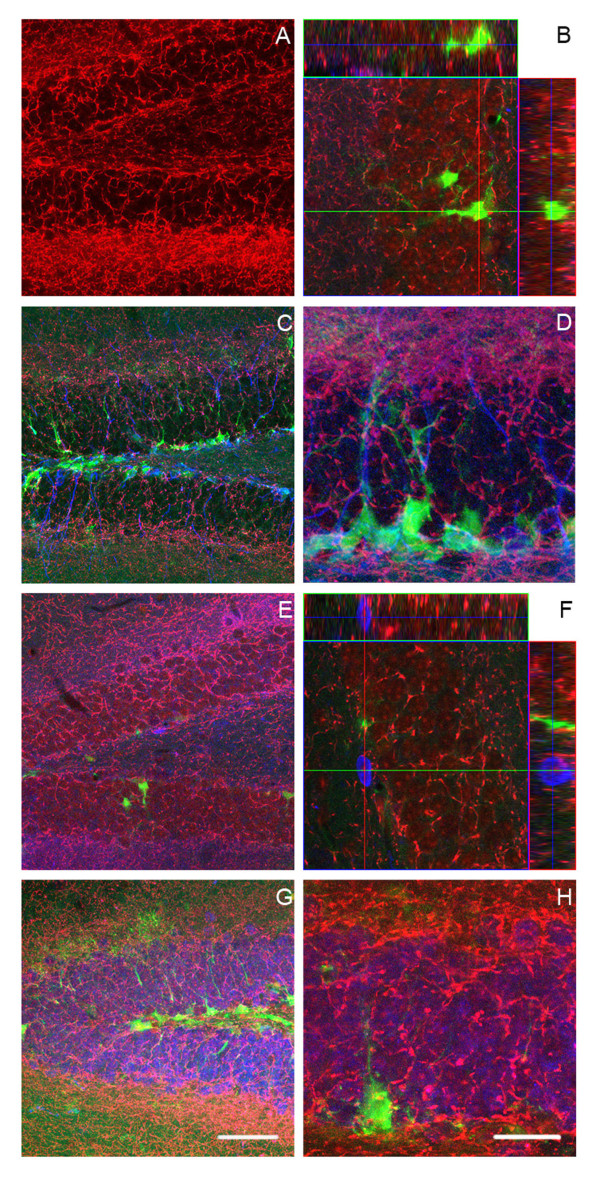
**Cannabinoid receptor 1 immunoreactivity in the dentate gyrus**. We here show representative photomicrographs of immunofluorescent staining of 40 μm thick mouse brain sections. CB1 immunoreactivity appears in red, Nestin-GFP in green, DCX, Calretinin (CR) and NeuN in Blue. The confocal scanning photomicrograph, 1 μm thickness, 40× magnification, shows that the CB1 receptor (red) is highly expressed in the dentate gyrus (A). The three-dimensional reconstruction of z-series of 8 confocal scanning photomicrograph (1.5 mm each) in shown in (B). The co-localization with Nestin-GFP (green) was found in some of cells with rounded morphology, less in the radial glia-like type-1 cells as shown in detail using orthogonal projections. The projection of 11 (C) and 13 (D) confocal scanning micrographs (1,5 μm thickness) reveals that DCX-positive cells (blue) show co-localization with the CB1 receptor (red). The 113× magnification shows in more detail the expression of CB1 receptor in DCX-positive cells (D). The confocal scanning photomicrograph, 1 μm thickness, magnification 40× shows a co-localization of Calretinin-positive staining with CB1 receptor (E). The three-dimensional reconstruction of z-series of 6 confocal scanning photomicrographs (1.5 mm each), 113× magnification show that Calretinin-positive cells (blue) are surrounded by CB1 receptor (red). The three-dimensional reconstruction of z-series of 13 (G) and 9 (H) confocal scanning photomicrographs (1.5 μm each) show immuno-reactivity to CB1 (red) in NeuN (blue) cells. The 113× magnification shows the co-localization in more detail (H). The representative scale bar is in G (50 mm) for A, C, E, and G; it is in H (20 mm) for B, D, F, and H.

Based on this model of adult hippocampal neurogenesis and the sequence of cellular morphology and marker expression, we found CB1-receptor expression spread over the entire dentate gyrus and the whole course of neurogenesis (Fig. 4A). However, it appeared that comparatively less staining was observed in the population of Nestin-positive type 1 cells (Fig. 4B) and type 2a cells. However, there was a tendency towards a stronger signal in cells expressing DCX (type 2b/3 cells; Fig. 4C, D), postmitotic new neurons that express Calretinin (Fig. 4E, F) and NeuN-positive mature new neurons (Fig. 4G, H). This implies that CB1 expression would increase with the degree of differentiation from type-2b cells onwards.

### CB1 mRNA expression is induced by activity

**Figure 5:**
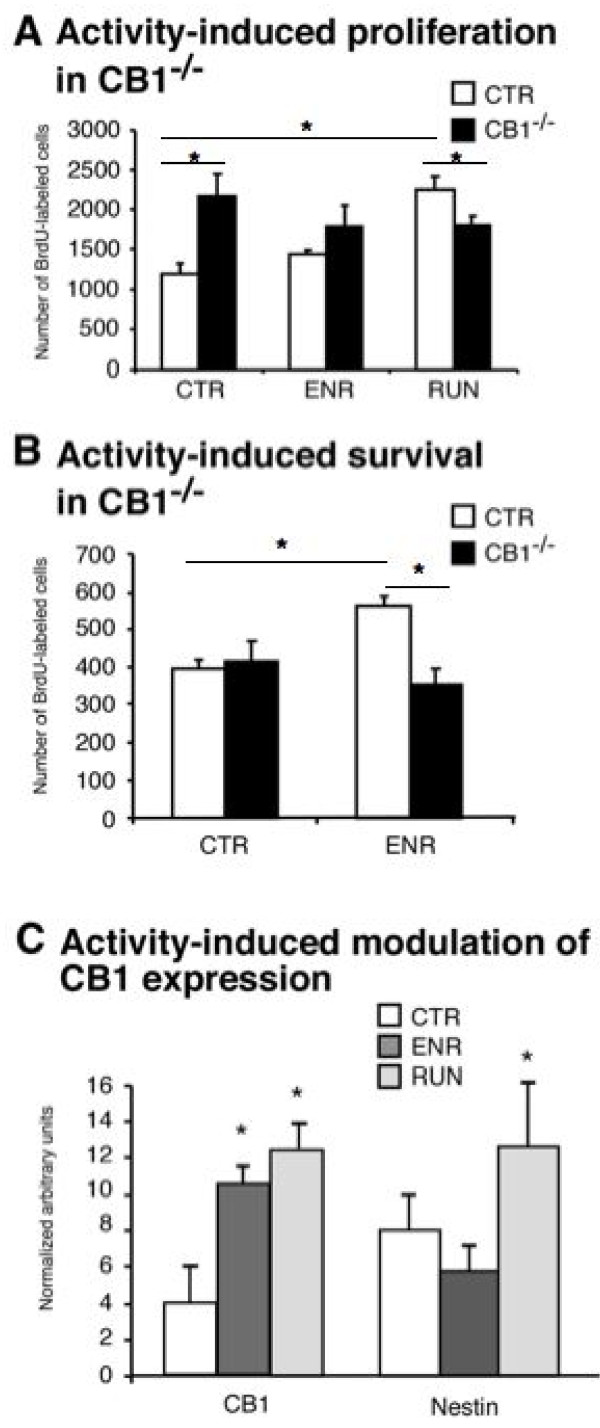
**Activity induced effects**. Running is known to enhance proliferation of cells in the dentate gyrus while enriched environment has a stronger effect on neuronal survival. We found that while RUN increased cell proliferation in WT mice, this was not the case in CB1^-/- ^mice. CB1^-/- ^mice showed an increased baseline proliferation (A). In CB1^-/- ^ENR-induced increase in cell survival was abolished. (B) Both housing conditions resulted in an increase of CB1-mRNA in the hippocampus of wild type mice. The early neuronal marker Nestin was only increased in the RUN paradigm (C); * p ≤ 0.05.

We subjected adult untreated female C57Bl/6 mice to either voluntary wheel running (RUN) or enriched environment (ENR). One group was housed in conventional cages (CTR). Type-2 cells are highly regulated cells *in vivo *and are influenced by behavioral activity. We have previously shown that both environmental enrichment and voluntary physical activity induce adult hippocampal neurogenesis while having differential effects on the type-2 progenitor cells [40]. We here confirmed this observation at the level of Nestin-mRNA expression, showing that RUN but not ENR increased Nestin mRNA (Fig. 5C CTR vs. ENR: 8 ± 2.2 vs. 6 ± 1.7; n = 6; p = 0.214; CTR vs. RUN: 8 ± 2.2 vs.13 ± 5.1; n = 6; p = 0.041). These findings are consistent with the counts of BrdU positive cells (Fig. 5A CTR vs. ENR: 1184 ± 81 vs.1462 ± 35; n = 5; p = 0.051; CTR vs. RUN 1184 ± 81 vs. 2197 ± 94; n = 5; p = 0.002). In the same samples, we found that both RUN and ENR increased the expression of CB1 receptor mRNA in the hippocampus (Fig. 5C; CTR vs. ENR: 4 ± 1.8 vs.10.5 ± 0.8; n = 6; p = 0.0001; CTR vs. RUN: 4 ± 1.8 vs. 12.5 ± 1.6; n = 6; p = 0.0001). This supported our result from immunohistochemistry with regard to the expression of CB1 on neuronal progenitor cells. We next wanted to know, whether CB1 receptor expression would also be necessary to elicit the effects of RUN and ENR on adult neurogenesis

### Activity-induced neurogenesis is absent in CB1^-/- ^mice

**Figure 6:**
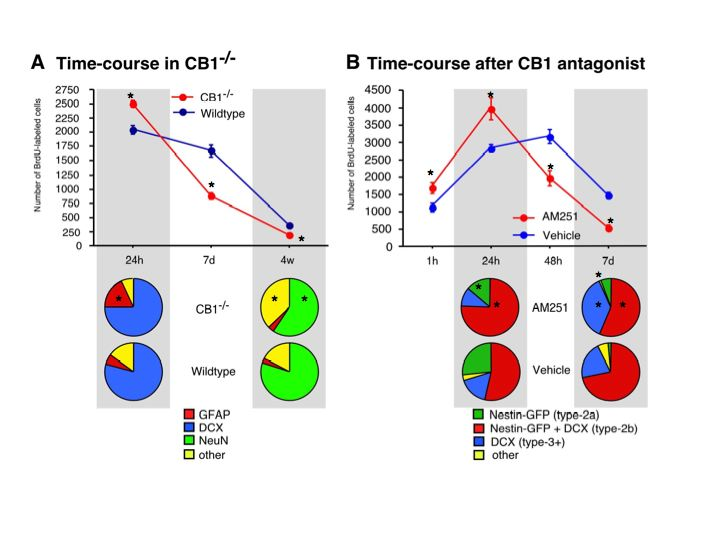
**Effects of CB1 absence on neuronal maturation**. By using animals at different points of time after BrdU injection a detailed time course of neuronal maturation has been established. At 24 h after BrdU the number of BrdU-positive cells was increased in the mutant mice but at 4 weeks the number of BrdU-positive cells was reduced compared to the controls. The percentage of NeuN-positive cells was also reduced resulting in a net reduction of adult neurogenesis in the mutants. When we looked at the 24 h time point we found a relative reduction in the number of proliferative DCX-positive cells At an intermediate 7d time point, the increased proliferation in CB1^-/- ^animals had yielded to a strong reduction in BrdU positive cells compared to controls (A). To investigate early stages of neuronal maturation, we used Nestin-GFP-reporter mice and injected the CB1 antagonist AM251. At 1 h after BrdU application counts of BrdU-positive cells reflect S-phase entry. AM251-treatment increased the number of BrdU-positive cells compared to vehicle controls. At 24 h the numbers had roughly doubled, reflecting a completed cell cycle. Phenotypic analysis revealed that this increase was largely accounted by type-2b cells and later DCX-positive cells, whereas type-2a was even reduced. At 48 h control values for BrdU began to be higher than in the AM251-treated mice, leading to an almost two-fold reduction in AM251-treated mice at 7d (B); * p ≤ 0.05.

We next subjected female CB1^-/- ^mice and their littermates to voluntary wheel running (CB1^-/-^/RUN and WT/RUN) for 10 consecutive days. We found that voluntary wheel running did not increase cell proliferation in CB1^-/- ^mice as it did in WT mice. CB1^-/- ^mice however, showed an increased baseline proliferation consistent with the findings presented in figure 6A (Fig. 5A; WT/CTR vs. CB1^-/-^/CTR: 1184 ± 81 vs. 2251 ± 118; n = 5; p = 0.002; WT/RUN vs. CB1^-/-^/RUN: 2197 ± 74 vs. 1772 ± 68; n = 5; p = 0.018). ENR primarily affects cell survival. In CB1^-/- ^mice the ENR-induced increase in cell survival was abolished (Fig. 5B; WT/ENR vs. CB1^-/-^/ENR: 553 ± 14 vs. 362 ± 42; n = 5; p = 0.023). Taken together both results suggest that CB1-mediated mechanisms play an important role in mediating the behavior-induced regulation of adult hippocampal neurogenesis.

### Proliferation is increased but neurogenesis is reduced in CB1^-/- ^mice

We now returned to evaluation of CB1 effects during the course of adult neurogenesis and how the affected neuronal maturation stages are influenced via the cannabinoid-mediated pathway. We therefore conducted a time-course study in female untreated CB1^-/- ^mice compared to littermate controls (WT). At 24 h after BrdU the number of BrdU-positive cells was higher in the mutant mice (WT vs. CB1^-/-^: 2175 ± 32 vs. 2489 ± 27; n = 5; p = 0.0042; Fig. 6A) but was lower at 4 weeks after BrdU (WT vs. CB1^-/-^: 368 ± 21 vs.180 ± 15; n = 5; p = 0.0024; Fig. 6A). The percentage of NeuN-positive cells was also lower at 4 weeks after BrdU resulting in a net reduction of adult neurogenesis in the mutants (WT vs. CB1^-/-^: 82% vs. 66%; n = 5; p = 0.048; Fig. 6A). When we looked at 24 h we found a relative reduction in the number of proliferative DCX-positive cells in the knock out animals (WT vs. CB1^-/-^: 81% vs. 75%; n = 5; p = 0.063; Fig. 6A). The contribution of type-2 cells to the increase in proliferation in CB1^-/- ^mice could not be further elucidated. GFAP-positive cells largely accounted for the initial increase in proliferation (WT vs. CB1^-/-^: 6% vs. 20%; n = 5; p = 0.001; Fig. 6A). Additional studies will have to investigate to what degree these GFAP-positive cells include the radial glia-like type-1 cells. At an intermediate point in time at 7 days after BrdU injection, the proliferation in CB1^-/- ^animals was strongly reduced compared to controls, leading to the later reduction in adult net neurogenesis at 4 weeks after BrdU injection (WT vs. CB1^-/-^: 1683 ± 65 vs. 836 ± 19; n = 5; p = 0.002; Fig. 6A).

### CB1 receptor antagonist AM251 induces cell proliferation of DCX-positive precursor cells and reduces further differentiation

These observations suggested that CB1-activity stimulates neurogenesis in particular from type-2 cells onward, especially affecting the DCX-positive populations by accelerating development and promoting survival. To further study this shift from reduction to stimulation, which is already apparent in the time-course depicted in Fig. 6A, we conducted another experiment in untreated adult female Nestin-GFP-reporter mice to identify effects of CB1 antagonist AM251 on the intermediate precursor cell stages.

At 1 h after BrdU application counts of BrdU-positive cells reflect S-phase entry. As expected, AM251-treatment increased the number of BrdU-positive cells compared to vehicle controls (vehicle vs. AM251: 1142 ± 28 vs. 1674 ± 27; n = 5; p = 0.038; Fig. 6B). At 24 h the numbers had roughly doubled, reflecting a completed cell cycle. The relative difference between the groups was maintained at this point in time, confirming that CB1 receptor activity reduced cell proliferation from preventing S-phase entry onward (vehicle vs. AM251: 2810 ± 35 vs. 4048 ± 120; n = 5; p = 0.0042; Fig. 6B). Phenotypic analysis revealed that this increase was largely accounted by type-2b cells, whereas type-2a was even reduced (vehicle vs. AM251: type-2b 58% vs. 77%; n = 5; p = 0.032; type-2a 27% vs. 12%; n = 5; p = 0.024; Fig. 6B).

At 48 h AM251 values for BrdU began to be lower than in the vehicle-treated mice (vehicle vs. AM251: 3182 ± 62 vs. 2035 ± 78; n = 5; p = 0.028; Fig. 6B), leading to an almost two-fold reduction in AM251-treated mice at 7d (Fig. 6B; vehicle vs. AM251: 1627 ± 18 vs. 567 ± 15; n = 5; p = 0.023). Most likely the BrdU positive cells in later maturation stages (depicted as "other") mainly account for the decrease (vehicle vs. AM251: other 3% vs. 0.5%; n = 5; p = 0.002; Fig. 6B).

## Discussion

In this study we have found substantial differences between THC and CBD treatment, supporting the previously reported disruption of memory formation by THC [41]. We did not find a suggestive association between CBD-mediated CB1 activity, learning performance in the water maze, and adult hippocampal neurogenesis. THC impaired cognitive and enhanced locomotor function but had no effect on neurogenesis, when given chronically. The learning phenotype in the Morris water maze did not correlate with the neurogenesis phenotype. Taken together, both THC and CBD effects on this type of hippocampus-dependent function cannot be linked to adult neurogenesis in a straightforward way. This discrepancy between functional and cellular hippocampal features had not yet been shown for THC or CBD, but the phenomenon of divergence between learning paradigms and neurogenesis is known from other studies (reviewed in [42]). When neurogenesis was blocked by focal x-radiation the mice that had been exposed to an enriched environment still performed better in the Morris water maze than the mice housed in standard cages. Since enriched environment enhances the survival of newly generated neurons, the investigators claimed separate effects of the enriched environment on neurogenesis and on spatial learning [43]. Other groups showed that hippocampal irradiation immediately before the test had no effect, while irradiation days before the test impaired long-term memory in the water maze, indicative of a critical time window [44].

We showed that CBD increased neurogenesis at the survival stage 4 weeks after BrdU injection. Similar to the neuronal survival effect of CBD it has been reported, that the synthetic non-selective cannabinoid agonist WIN-55,212-2 restored the physiologically decreased levels of adult neurogenesis in aged rats [45]. In the current study we have studied young adult mice, but it might be worthwhile to investigate in further studies the CBD effect in aged animals. CBD is known to be beneficial in schizophrenia or schizophrenic-like behaviour [17], where patients show a decrease in hippocampal volume and neurogenesis might be impaired [17,46]. CBD has some antipsychotic properties [47]. Moreover, smoking some strains of cannabis containing relatively more CBD, in addition to THC, appears to be more protective against the psychotic symptoms induced by THC alone [48].

A study by Boucher et al. has shown that THC impaired spatial memory and reversal learning, even in animals that received a THC pretreatment, indicating that although tolerance to the effects of THC on neuronal activity in the prefrontal cortex was reported, cannabinoid-induced memory impairment in these animals persisted [7]. Although we could only test our animals at one time point, including the information of tolerance resistance to THC from the reference mentioned above makes us confident that no acute or tolerance effects were present during our testing phase.

Taken together, the findings suggest diverse effects of the cannabinoid system on memory and cellular plasticity. These effects cannot be plainly categorized into impairing or enhancing effects of cannabinoid activation or deactivation [49]. The same might be true for the finding that THC increased the performance in the rotarod test. CB1 activation in the cerebellum by intra-cerebellar THC injection led to locomotor deficits [50]. Moreover, stimulation of cerebellar CB1 receptors with the agonists CP55,940 and HU-210 impaired rotarod performance [51]. THC injected intraperitoneally on the other hand failed to provoke motor coordination disturbances in wild type B6/CBA mice [52]. The route of administration seems to be a key difference between this one and the other studies. CB1 receptors were activated in all relevant brain regions and the local concentration of THC in a given brain structure was lower than when administered directly into the cerebellum [52]. In our study the mice took up the THC via the food, which led to improved rotarod performance. In the light of therapeutically targeting locomotor dysfunction with cannabinoids this finding might be notable. The therapeutic potential of the cannabinoids was also investigated in neurological diseases such as multiple sclerosis, Gilles de La Tourette syndrome, Parkinson and Huntington disease that all include locomotor disabilities [53]. Although the efficacy was not always clearly established, the undesirable effects observed were generally mild and well tolerated [54]. The drugs used to treat symptoms of multiple sclerosis (Sativex, contains THC and CBD) failed to change the neuropathological hallmarks of the disease. Patients reported only minor changes in memory loss, while improvements in locomotor and spasticity and neuropathic pain were dominant [55]. Experiments with THC and CBD in different concentrations might help to unravel the complex pattern of such treatments and should, as our results suggest, include measures of adult neurogenesis.

The neurogenic effect of CBD was not found in CB1^-/- ^animals. Although CBD has low affinity to CB1 and its effects are often mediated via non-CB receptors (e.g. the vanilloid receptor) at least three other studies support that cannabidiol effects were CB1 receptor-dependent [19,56,57]. It might not be the only or usual mode of action, but with regard to enhanced adult hippocampal neurogenesis, CBD at least partially acts through the CB1 receptor. This result prompted us to investigate CB1 dependent regulation of neurogenesis utilizing CB1 receptor knock out animals as well as the CB1 receptor antagonist AM251 in Nestin-GFP-reporter mice.

In female CB1^-/- ^mice on a C57BL/6 background we found increased proliferation 24 h after BrdU injection and decreased net-neurogenesis (7 days and 4 weeks after BrdU injection). Jin and colleagues reported impaired progenitor cell proliferation in CB1^-/- ^mice but found contradicting increases with pharmacological CB1 antagonists SR141716A and AM251 [30,58]. We did not observed such discrepancy. The time point of analysis in the Jin et al. study was 3 days after BrdU injection. Thus, they might not have detected the most acute effects. Using the same compound AM251 on wild type mice, we got different results at 7 days after BrdU injection. Although we observed an increase of BrdU-labeled cells at 1 h and 24 h, which would be in line with the findings by Jin et al., we found a decrease in BrdU-labeled cells at 48 h and 7 days. When phenotyped, DCX-positive cells accounted for the increase in proliferation at early time points, but at late time points fewer DCX- and more Nestin-positive cells were present indicating that maturation was impaired at the DCX-stage. This supports our data, that CB1 stimulation or blockage had different effects on neuronal progenitor proliferation and differentiation or maturation. The same pro-proliferative effect of AM251 at 24 h after BrdU injection have also been observed by Hill et al. in rats [59].

One notable difference between the study of Jin et al. and other studies (including ours) was their use of a CB1^-/- ^strain bred onto the CD1 background [60]. We have previously shown that CD1 show a very unusual pattern of baseline adult neurogenesis. Despite lower levels of proliferation compared to C57BL/6 they actually achieve high levels of net neurogenesis since survival exceeds any other strain investigated so far [61]. Another difference between the studies might have been the use of male vs. female mice since a recent report demonstrated differences in CB1 receptor abundance in the hippocampus between female and male mice [62]. Unfortunately Jin et al. did not report the gender examined in their studies. On the other hand, receptor abundance per se does not allow strong conclusions about receptor activity.

In addition, we show that the time point of measurement is critical when assessing the effects of the antagonists. Our findings imply, that CB1 receptor activity would increase proliferation of type-1/2a, reduce proliferation of type-2b/3 but accelerate maturation from these cells and lead to a net reduction of adult neurogenesis. Consequently, the increase observed in the Jin et al. study after 3 days of AM251 in parallel to 3 days of BrdU is likely to actually reflect a mix of increases and decreases, which can only be untangled with a different BrdU injection protocol and a distinction of the different precursor cell types.

CB1 receptors are expressed in the course of neuronal development but they are present on all precursor cells, beginning with the radial glia-like type-1 cells [23]. CB1 expression appears to increase with differentiation, an observation that has also been made in embryonic cortical development [63]. Together with our previous data on wild type mice [40] these data indicate that CB1 is expressed by cells that are primarily affected by activity-dependent regulation of adult hippocampal neurogenesis. We could consequently show that this type of regulation is impaired, if the CB1 receptor is absent. Keeney at al. have shown that the CB1 antagonist Rimonabant (SR141716) decreased running activity in C57Bl/6 female mice when injected for 9 consecutive days at the peak of running [64]. The situation in the knock out animal in our study is different, since CB1 is absent constitutively and not only at the peak of running like in the Keeney et al. study. It is also notable that SR141716 has different effects on neurogenesis than the absence of CB1 [30]. We measured running performance as the distance run per day for 10 consecutive days. As long as running the same distance is indicative of a similar stimulus for neurogenesis, the conditions should have been the same for CB1^-/- ^and wild type mice. In the hippocampus of wild type mice we found an upregulation of CB1 receptor mRNA in the ENR and RUN mice along with an increase in Nestin mRNA only in the RUN paradigm. This is in line with studies reporting an increase in density of CB1 receptors in the hippocampus after voluntary wheel running. When AM251 was administered, activity-induced neurogenesis was impaired [65]. This result also supports our findings that activity-induced neurogenesis is absent in CB1^-/- ^mice. In contrast, a study using male mice that ran over a period of 6 weeks, CB1^-/- ^animals covered less distance but showed greater numbers of DCX-expressing cells in the dentate gyrus indicating that a running-phenotype can be discovered after a prolonged running period [66]. Another study reported that CB1 receptor sensitivity in the striatum increased after voluntary wheel running [67]. In the light of therapeutic interventions targeting the cannabinoid system, increasing the receptor by simple running might be of interest.

The putative contribution of new neurons to hippocampal function has recently become increasingly clearer. Neurogenesis and specific aspects of learning (temporal separation, contextual integration, flexibility of relearning, and integration of novelty) [34,68,69] have been linked and a role in affective behavior has been described [70,71]. Jiang and colleagues have suggested that the CB1-mediated effects of HU210 on adult neurogenesis might have anxiolytic and anti-depressant-like consequences [25]. It might thus be that the cannabinoid-dependent regulation of adult neurogenesis is more relevant for the emotional than for the cognitive aspects of hippocampal function.

## Conclusions

In this study we have shown that (1) exogenous cannabinoids THC and CBD differ in their effects on spatial learning and adult neurogenesis. (2) CBD did not impair learning but increased adult neurogenesis despite (3) a CBD-induced reduction in cell proliferation. We found (4) the pro-neurogenic effect of CBD to be dependent on the CB1 receptor, which (5) shows a widespread expression over the entire dentate gyrus, including the neuronal precursor cells. Similarly, (6) the pro-neurogenic effect of environmental enrichment and voluntary wheel running depended on the presence of the CB1 receptor. Along the same line, (7) voluntary wheel running increased CB1 receptor mRNA in the hippocampus. We observed that (8) in the absence of CB1 receptors, cell proliferation was increased and neuronal differentiation reduced.

Although it has been reported that CBD binds with a low affinity to the CB1 receptor, its mode of action on neurogenesis seems to involve the CB1 receptor since CBD had no effect on CB1^-/- ^animals. This prompted us to investigate the CB1 dependent regulation of neurogenesis using a genetic model and an antagonist. Taken together, our results indicate that the CB1 receptor appears to play an important role in modulating adult hippocampal neurogenesis. More specifically, CB1 affects the stages of adult neurogenesis that involve intermediate highly proliferative progenitor cells (type-2 and type-3 cells) and the survival and maturation of the new neurons. While these results are mostly in line with previous results on CB1 function in adult neurogenesis (reviewed in [13]), they also go beyond what was known since our data elucidate the time-course of this action and reveal a contribution of CB1 to activity-dependent regulation. Although others and we found CB1 receptor expression on precursor cells, the effects on cannabinoids on neurogenesis might still be indirect as well.

## Materials and methods

### Animals

The generation of CB1-/- mice on a C57Bl/6 background has been described elsewhere [72]. The animals were kindly provided by Roland Martin, National Institutes of Health, Bethesda. The control group consisted of age-matched littermates. Since we carried out heterozygote breeding, we genotyped the progeny by PCR using the following primers

3'AAGAACGAGATCAGCAGCCTCTGTT5'; 3'GGATTCAGAATCATGAAGCACTCCA5'.

The experiments measuring the early stages of neurogenesis were performed in transgenic mice expressing the green fluorescent protein (GFP) driven by regulatory elements of the Nestin gene, Nestin-GFP mice [73].

All the animals were held in the same room with a consistent 12-hour-light-dark-cycle and were fed with the standard or supplemented food and water *ad libitum*. To estimate the daily food intake, animals and food were weighted every 3^rd ^day for the whole period of the experiment (see additional file 1, 2).

All applicable local and federal regulations on animal welfare were followed. The animal protocol was approved by "Landesamt für Arbeitsschutz, Gesundheitsschutz und technische Sicherheit Berlin (LaGetSi)".

### Experimental design

Thirty female CB1^-/- ^and their littermates (WT) mice were randomly assigned to either enriched (CB1^-/-^/ENR; WT/ENR) or standard housing (CB1^-/-^/CTR; WT/CTR) or standard cages that were equipped with a running wheel (CB1^-/-^/RUN; WT/RUN). RUN-assigned animals had unlimited access to the running wheel (Tecniplast, Hohenpeißenberg, Germany) for 10 days. The enriched housing in which the animals lived for 4 weeks was similar to our previous studies [74]. Briefly, it consisted of a spacious cage of approximately 80 × 80 cm floor area, complemented with a re-arrangeable system of tubes, a cardboard box house and a crawling ball. The ENR and CTR animals were housed in groups of 5, in the RUN cages 2 animals lived together. To evaluate CB1 mRNA expression changes by activity, we subjected additional 5 female C57Bl/6 to either experimental condition (RUN, ENR, CTR).

In a different set of experiments, 30 female wild-type mice were housed in standard cages and fed with either a diet supplemented with THC or CBD or without any drug (CTR) for 6 weeks (chronic treatment). Ten CB1 knockout mice were fed with either a diet supplemented with CBD or standard food.

After treatment 10 animals of each group were tested in the Morris water maze for spatial memory performance and in the rotarod for locomotor functions. All remaining mice received daily intraperitoneal injections of Bromdesoxyuridin BrdU (50 μg/kg body weight, Sigma) for either 1 day or 5 consecutive days and were killed either 24 hours (proliferation) or 4 weeks (survival) after the last BrdU injection. At the starting point of all experiments the age was 6-8 weeks. The behavioral testing occurred 6 weeks later so that the animals were at least 12 weeks old when the testing started and 16 weeks when the survival time point of adult neurogenesis was assessed.

To analyze the chronic impact of the cannabinoids on the early stages of neuronal development, we utilized transgenic animals where the Nestin promotor is linked with an eGFP-construct emitting green fluorescence [73]. Twenty Nestin-GFP female animals were fed for 6 weeks with a THC-rich or CBD-rich or standard diet (see below). The antagonist AM251 compared to vehicle injections (Torisolve, Tocris) was used on 10 Nestin-GFP-reporter mice to evaluate the impact of CB1 at the early stages of neurogenesis, as described previously [36]. In parallel, five female CB1^-/- ^and their littermates (WT) each received BrdU for either 1 day or 5 consecutive days and were killed either 24 hours (proliferation), 7 days or 4 weeks (survival) after the last BrdU injection

### Cannabinoid and antagonist treatment

THC-rich or CBD-rich plant extract was kindly provided by GW-Pharmaceuticals, UK. The plant extracts were incorporated into a standard diet by ResearchDiet, USA at the concentration of either 41.2% for active THC or 38.8% for active CBD. The diets were colour -labelled for easier handling.

CB1 antagonist AM251 (Tocris) was injected intraperitoneally at 0.25 mg/kg in Tocrisolve (0.5 μg/μl in 100 μl per animal).

### Immunohistochemistry

Animals were deeply anesthetized with ketamine and perfused transcardially with cold 4% paraformaldehyde in 0.1 M phosphate buffer, pH 7.4. The brains were dissected from the skulls and were postfixed overnight. Before sectioning from a dry-ice-cooled copper block on a sliding microtome (Leica, Bensheim), the hemispheres were transferred to 30% sucrose in 0.1 M phosphate buffer, pH 7.4, until they had sunk. Hemispheres were cut in the coronal plane in 40 μm thick sections and cryo-protected. The level of generation of new cells was determined by the *in vivo *injection of BrdU, which incorporates during the S-phase into the cell and thus labelled proliferating cells. BrdU labelled cells in the subgranular zone of the dentate gyrus in the hippocampus were quantified as described previously [69]. Briefly all labelled cells per dentate gyrus were counted in every 6^th ^section containing the hippocampus. For each animal 9 sections have been counted (both sides). The number was multiplied by six to estimate the total cell number per brain. For BrdU staining, DNA was denatured in 2N HCL for 30 minutes at 37°C. Free-floating sections were than rinsed in 0.1 M borate buffer, pH 8.5, and thoroughly washed in tris-buffered saline (TBS), pH 7.4. To block endogenous peroxidase reactions, sections were pre-treated with 0.6% H_2_O_2_. The rat-anti-mouse-BrdU antibody (Harlan Seralab) was diluted 1:500 in TBS supplemented with 0.1% TritonX-100, 0.1% Tween 20 and 3% donkey serum (TBS-plus) and the sections were incubated overnight at 4°C. After rinsing the sections in TBS and a blocking step in TBS-plus, an incubation step with the biotinylated secondary antibody (donkey-anti-rat, Vector) diluted 1:500 in TBS-plus followed. ABC reagent (Vectastain Elite, Vector Laboratories) was applied for 1 h at a concentration of 9 μl/ml for each reagent. Diaminobenzidine (DAB, Sigma) was used as a chromogen at the concentration of 0.25 mg/ml in TBS with 0.01% H_2_O_2 _and 0.04% nickelchloride followed by rinsing with tap water and TBS. The sections were mounted on slides and coverslipped with Neomount. To phenotype the proliferating cells, we used triple staining for BrdU and a combination of maturation markers as applicable. A total of randomly selected 50 BrdU-positive cells per animal were phenotyped. Knowing the absolute number of BrdU cells in a given brain, we were able to convert the percentage of cells expressing one of the maturation markers and BrdU into the absolute number of cells per phenotype in the whole brain. All counting were done blinded by the same researcher as described previously [38,69]. The primary antibodies were applied in the following concentrations: BrdU (1:500, Harlan Seralab), anti-Doublecortin (DCX, 1:200, Santa Cruz Biotechnologies), anti-Calretinin (1:250, Santa Cruz), anti-GFP (to visualize Nestin, 1:500, Swant), anti-NeuN (1:100, Chemicon), anti-GFAP (1:250, Chemicon), anti-CB1 (1:250, LifeBioscience). Secondary antibodies were anti-goat, anti-rabbit, anti-mouse, and anti-rat (1:250, Jackson Laboratories) directly coupled to a fluorochrome for confocal analysis. To test for statistical significant differences (p = 0.05) between two groups, we used the non-parametrical Mann-Whitney-U-test.

### RNA isolation and RT-PCR

RNA was isolated with an RNeasy mini isolation kit according to the manufactures instructions (Qiagen, Hilden, Germany). CB1 and Nestin content in 1 μg RNA per sample was measured using the QuantiFast SYBR Green RT-PCR Kit according to the manufactures instructions (Qiagen, Hilden, Germany). We used the following primer pairs generated with primer3 software: CB1: forward CTGGTTCTGATCCTGGTGGT, reverse TGTCTCAGGTCCTTGCTCCT; Nestin: forward TTGAGGCCTCCAGAAGAAGA, reverse GCCATCTGCTCCTCTTTCAC. The RNA amount was normalized to the housekeeping gene GAPDH. Statistical analysis has been done using the non-parametrical Mann-Whitney-U test between two groups. PCR was performed using an OPTICON II (BioRad, Munic, Germany).

### Behavioral Tests

The Morris water maze (MWM) test is widely used to test rodents for spatial memory performance [75]. We followed the protocol revised by Wolfer and Lipp [76]. Six trials of training, each maximally lasting 2 minutes, were given each day. Latencies to reach the platform and swim paths were recorded with an automatic video tracking system (Ethovision, Noldus, Utrecht, Netherlands).

Animals were exposed to the MWM that contained an escape platform submerged 1 cm below the water line. The platform was kept at a constant location within the pool during the first 3 days of training. On the morning of the 4^th ^day the escape platform was placed in the quadrant opposite to the first target quadrant to start the reversal learning task for two more days. The first trial of the reversal period was analyzed as "probe trial". To control for parameters that are not hippocampus-dependent such as vision impairments, the task was afterwards repeated with a visible platform. To evaluate learning of the spatial location of the platform, latencies to reach the platform (in seconds) and total length of swim path (in pixels converted to cm) were compared between trials. Additionally, the time spent in the target quadrant on the probe trials was used as an indicator of targeted searching for the platform. During the reversal learning, time spent in quadrant 1 (location of the platform during initial training) versus quadrant 3 (location of the platform during reversal training) was measured.

To analyze performance in the MWM test, we performed a repeated measure ANOVA test of the daily means. Analysis of the differences between the groups in the parameters escape latency, and distance moved per day, using the Fisher post-hoc-test, if applicable.

To test general locomotor functions and fitness of the animals, a rotarod was used. The mice were placed on a slowly rotating rod (20 rpm) and a stopwatch was started. The rod accelerated with 20 rpm. When the mice overbalanced and touched the ground, the stopwatch stopped automatically. Each animal performed 4 trials.

## Competing interests

The authors declare that they have no competing interests.

## Authors' contributions

SAW has designed the study, carried out the in vivo experiments, did statistical analysis and drafted the manuscript

ABS participated in the design of the study and did the behavioral analysis

KF and PLG carried out the CB1 staining and prepared Fig. 4

ST revised the manuscript

AM carried out confocal analysis

TPW participated in the draft of the manuscript and carried out the immunoassays

GRR carried out cell culture work (data not shown)

AM carried out the RNA analysis

OU worked on the manuscript

GK supervised the experimental design, prepared figures and substantially contributed to the drafting, writing and revision of the manuscript

All authors read and approved the final manuscript.

## Supplementary Material

Additional file 1 **Weight gain and food intake**. The two graphs show the food intake and weight gain (g) during the whole period of the experiment of 6 weeks. In the beginning of the experimental period, some variances could be seen in the food intake between the groups at certain days. At 6 weeks, when the analysis started, all groups reached a similar level of food intake and weight in average. Click here for file

Additional file 2 **Weight gain and food intake**. The two graphs show the food intake and weight gain (g) during the whole period of the experiment of 6 weeks. In the beginning of the experimental period, some variances could be seen in the food intake between the groups at certain days. At 6 weeks, when the analysis started, all groups reached a similar level of food intake and weight in average. Click here for file
