# Supplementary figures and images for: Cannabinoid receptor CB1 mediates baseline and activity-induced survival of new neurons in adult hippocampal neurogenesis
Source: Cell Commun Signal. 2010 Jun 17;8:12. doi: 10.1186/1478-811X-8-12 (PMC2898685; doi:10.1186/1478-811X-8-12)

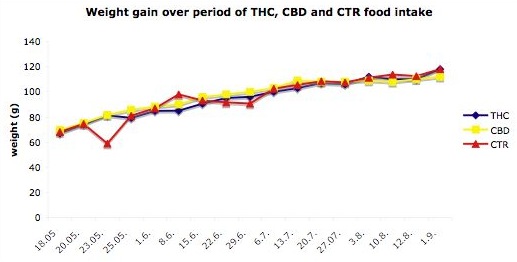

Supplement: Additional file 1 — Weight gain and food intake. The two graphs show the food intake and weight gain (g) during the whole period of the experiment of 6 weeks. In the beginning of the experimental period, some variances could be seen in the food intake between the groups at certain days. At 6 weeks, when the analysis started, all groups reached a similar level of food intake and weight in average. [file 1478-811X-8-12-S1.JPEG]

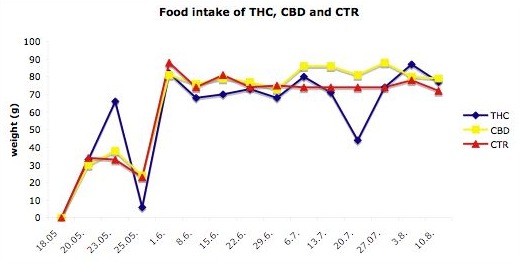

Supplement: Additional file 2 — Weight gain and food intake. The two graphs show the food intake and weight gain (g) during the whole period of the experiment of 6 weeks. In the beginning of the experimental period, some variances could be seen in the food intake between the groups at certain days. At 6 weeks, when the analysis started, all groups reached a similar level of food intake and weight in average. [file 1478-811X-8-12-S2.JPEG]
